# Frequency of team simulation and reduction in maternal deaths following Safer Births Bundle of Care implementation—a prospective observational study

**DOI:** 10.1186/s41077-025-00387-7

**Published:** 2025-11-14

**Authors:** Kjetil Torgeirsen, Benjamin Kamala, Estomih Mduma, Florence Salvatory Kalabamu, Robert Moshiro, Doris Østergaard, Jan Terje Kvaløy, Hege Langli Ersdal

**Affiliations:** 1 SAFER Simulation Center, Stavanger, Norway; 2 Faculty of Health Sciences, University of Stavanger, Stavanger, Norway; 3 Department of Research, Haydom Lutheran Hospital, Haydom, Tanzania; 4 School of Public Health, Muhimbili University of Health and Allied Sciences, Dar Es Salaam, Tanzania; 5 Department of Paediatrics and Child Health, Muhimbili University of Health and Allied Sciences, Dar Es Salaam, Tanzania; 6 Hubert Kairuki Memorial University, Dar Es Salaam, Tanzania; 7 Department of Paediatrics and Child Health, Muhimbili National Hospital, Dar Es Salaam, Tanzania; 8 Department of Clinical Medicine, University of Copenhagen, Copenhagen, Denmark; 9 Copenhagen Academy of Medical Education and Simulation, Capital Region of Denmark, Copenhagen, Denmark; 10 Department of Research, Section of Biostatistics, Stavanger University Hospital, Stavanger, Norway; 11 Department of Mathematics and Physics, University of Stavanger, Stavanger, Norway; 12 Department of Simulation-Based Learning, Stavanger University Hospital, Stavanger, Norway

## Abstract

**Background:**

Safer Births Bundle of Care (SBBC) is a continuous quality improvement (CQI) program, implemented in 30 facilities in Tanzania, resulting in a 75% reduction in maternal deaths. Simulation training was introduced as a component of the CQI efforts, targeting individual and team skills, focusing on identified clinical needs.

**Objective:**

The aim of this study was to describe the frequency of documented simulation sessions and the number of recurrent participants and associations with changes in maternal death.

**Methods:**

SBBC was a stepped-wedge cluster randomised implementation study in 30 facilities in 5 regions of Tanzania from 2020 through 2023. The SimBegin® facilitator training program was introduced to train facilitators and support implementation of a training cascade. Fifteen selected healthcare workers were trained in three levels of SimBegin® to become facilitators (level 1) and mentors (level 2). Eight were trained to become instructors (level 3). In total, 90 local facilitators were trained to review local clinical data, run simulation sessions, and document in logbooks. Clinical data were collected from patient files by independent data collectors and looped back to the facilities on a weekly basis. Training interventions were planned, conducted, and evaluated based on identified gaps. Output measures were the frequency of simulation sessions, the number of recurring participants, and maternal death within 7 days postpartum the following month.

**Results:**

Overall, 281,165 parturient women were included in this study. The SBBC implementation period was 24–32 months, and 1280 simulation sessions were documented. Maternal deaths declined from 240/100,000 births in the baseline to 60/100,000 after the start of SBBC. There was an association between the frequency of simulation sessions and the reduction in maternal deaths (23% reduction per each unit increase on the log scale, *P* = 0.0018), and between the number of recurring participants and the reduction in maternal deaths (16% reduction per each unit increase on the log scale, *P* = 0.0006).

**Conclusion:**

This study documents a significant and clinically relevant association between the frequency of and participation in simulation sessions and the reduction of maternal deaths the following month.

**Trial registration:**

SBBC main protocol ISRCTN Registry: ISRCTN30541755. Prospectively registered 12.10.2020.

**Supplementary Information:**

The online version contains supplementary material available at 10.1186/s41077-025-00387-7.

## Introduction

In 2020, an estimated 287,000 women died due to pregnancy and childbirth [1]. The burden of maternal deaths is high in Sub-Saharan African countries, where 70% of maternal deaths occur [2, 3]. For Tanzania, the reported numbers of maternal deaths were 104 per 100,000 live births in 2022 [4].

Bleeding after birth is the leading cause of maternal deaths.

Globally, about 14 million women suffer from postpartum haemorrhage (PPH) each year, primarily caused by uterine atony [1, 5]. The majority of PPH-related deaths are preventable by timely and appropriate management following the WHO recommendations [1].

WHO has described poor quality healthcare as a global concern. Several human factors contribute to reduced patient safety, like communication breakdown, ineffective teamwork, and cognitive bias [6, 7]. Simulation is recognised as an efficient training method to improve patient safety and as a component of continuous quality improvement (QI), targeting individual medical expertise skills—individual skills as well as social (communication, collaboration, and leadership) and cognitive (situation awareness and decision-making)—team skills [8–13]. In this study, the term simulation is used to describe facilitator-led, in situ team simulation training.

Common barriers for implementation of simulation-based training in healthcare are financial constraints, high burden of work, lack of simulation resources, and a need for more patient outcome-oriented evidence supporting simulation training [14–17].

Previous Safer Births studies in Tanzania have indicated that frequent individual and team simulation training are feasible in a low-resource context and can impact clinical behaviour and patient outcomes [18–20]. Nelissen et al. found a 38% reduction of PPH and improved clinical outcomes after a half-day obstetric simulation training [21]. Another study found a limited clinical impact of low-dose high-frequency newborn resuscitation skill training and demonstrated the need for frequent simulation sessions to improve outcomes [18]. However, a gap remains in the literature. To the best of our knowledge, no previous studies have examined the association between the frequency of training sessions and reductions in maternal mortality.

In 2021, the SBBC program was introduced in five resource-challenged regions in Tanzania, including 30 healthcare facilities. The bundle is a result of over a decade of multidisciplinary collaboration between institutions inside and outside Tanzania and consists of several components: clinical innovations and a strategy for sustainable and scalable team simulations and data-driven QI, aiming to improve the quality of care around birth and reduce perinatal and maternal mortality [22].

SBBC included almost 300,000 births over a 3-year period, and the recently published report documents a 75% reduction in maternal death within 7 days postpartum (from 240/100,000 to 60/100,000 births) after full implementation of the program [23].

## Materials and methods

The aim of this study was to describe the frequency of team simulations and the number of recurrent participants in the 30 facilities during SBBC implementation and to investigate the association to maternal death.

### Study design and participants

This study is part of the SBBC stepped-wedge cluster randomised controlled implementation trial, which ran from March 1, 2021, to December 31, 2023 [22].

The study population included all healthcare workers and parturient women in the 30 healthcare facilities in five regions of Tanzania with a high burden of newborn and maternal deaths [23].

The training component of the SBBC program aims to strengthen competencies in day-of-birth emergency care through frequent individual skill training and in situ facilitator-led team simulation training sessions.

### Study interventions

The SBBC program encompasses four main components: simulation training interventions, continuous QI efforts, innovative clinical tools, and systems for sustainability and scalability.

#### Training of facilitators and healthcare workers

Through the SimBegin® program, facilitators were trained to conduct simulation sessions for the team with reflection-based and structured debriefings, to identify learning needs and plan proper training interventions, and to train new facilitators. The SimBegin® program is designed to be highly scalable to increase accessibility and efficiency of simulation training. The goal is to establish a sustainable system for simulation training by training selected individuals to a level where they can train new facilitators. The SimBegin® program has a three-step design: level 1 (becoming a facilitator), level 2 (becoming a mentor), and level 3 (becoming SimBegin® course faculty) training, with practising of facilitator skills between each level [23, 24]. The implementation of SimBegin® in SBBC followed an implementation strategy and a training cascade model, aiming to stimulate frequent interprofessional simulation sessions at the facilities.

The flexibility of the SimBegin® program enabled us to conduct the initial training of the national facilitators online. Travelling restrictions due to the ongoing COVID-19 pandemic prohibited simulation experts from Norway to travel abroad.

**Fig. 1:**
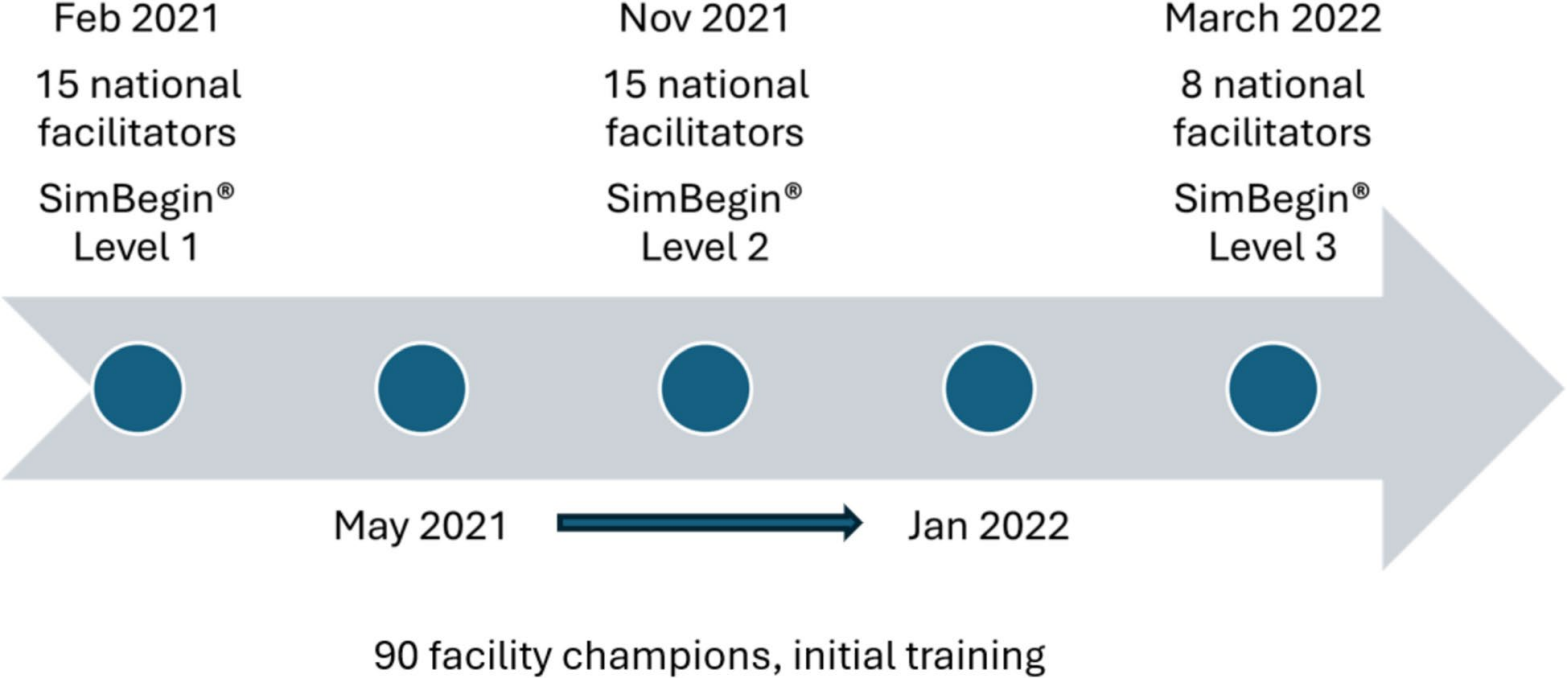
Training intervention timeline. Overview of the implementation of the SBBC training components intervention for national facilitators and local facility champions. The initial training consisted of a 12-day training program conducted at Haydom Lutheran Hospital in February 2021. The national facilitators were trained to a level of master trainers of the SBBC tools, the HBB, and the HMS BAB programs. Team simulation training was introduced through the SimBegin® level 1 training program by simulation experts from SAFER simulation centre. The simulation sessions used the following framework: brief, scenario, and a structured reflection-based debrief. In November 2021, the national facilitators completed SimBegin® level 2 training, enabling them to mentor facility champions responsible for frequent onsite team simulation sessions at the 30 SBBC facilities. In March 2022, eight of the national facilitators were selected and trained to a faculty/educator level, i.e. SimBegin® level 3, competent to train new facilitators in level 1 simulation methodology. Between May 2021 and January 2022, 90 selected facility champions (two–three from each facility) were trained by the national facilitators for 6 days at Haydom Lutheran Hospital. During these days, the facility champions underwent HBB and HMS BAB training, including theory and skill training, and the use of the innovative SBBC clinical tools. They also got an introduction to simulation training

Initially, 15 members from the Tanzanian Midwifery Association (TAMA), Pediatric Associations of Tanzania (PAT), and Association of Obstetricians and Gynaecology (AGOTA) were selected to become national facilitators (completed SimBegin® levels 2 and 3), responsible for cascading the SBBC trainings and mentoring local facility champions at the SBBC facilities (Fig. 1). The training of national facilitators and facility champions also included Helping Babies Breathe (HBB) and Helping Mothers Survive Bleeding after Birth (HMS BAB) training and the use of SBBC clinical tools [25–27]. Four pre-written scenarios were provided, two HBB newborn resuscitation scenarios and two HMS BAB scenarios. In addition, scenarios addressing the active management of the third stage of labour, eclampsia, and antepartum haemorrhage were designed during the study period.

#### Continuous quality improvement and the circle of learning

**Fig. 2:**
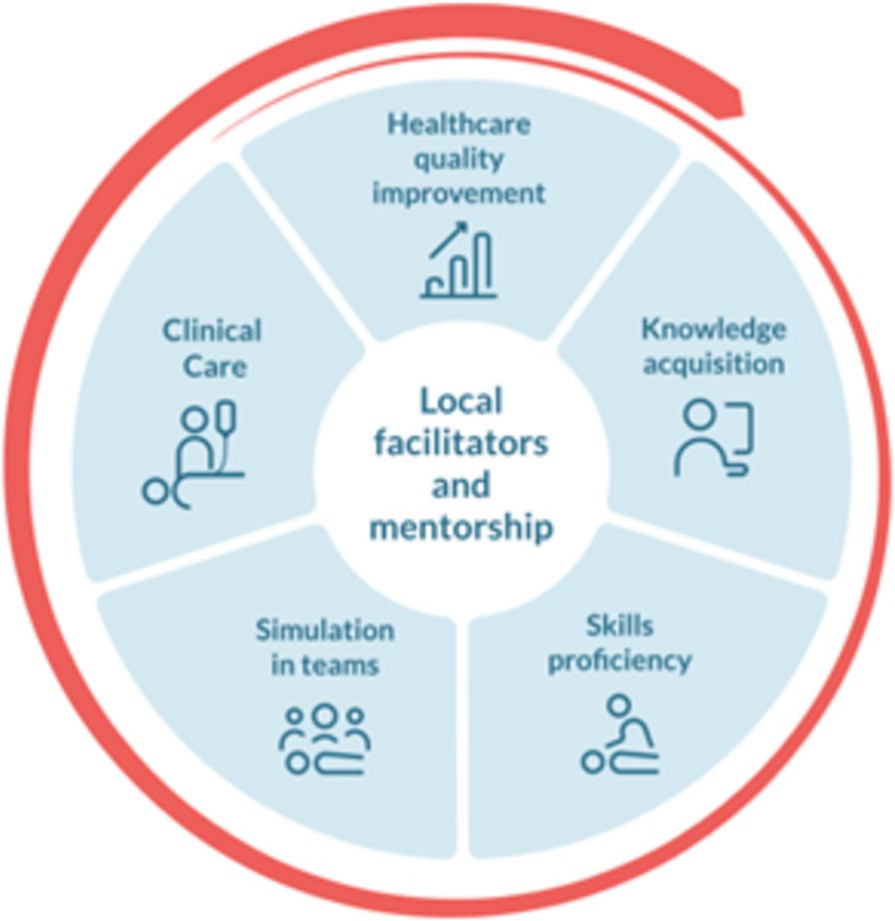
The Circle of Learning. The SimBegin program equips facilitators and mentors with tools to categorise clinical gaps and to plan training using the Circle of Learning. This framework focuses on five key areas: (1) Healthcare quality improvement, where SimBegin participants learn to identify gaps using clinical performance indicators. (2) Knowledge acquisition, ensuring healthcare workers have the necessary knowledge for clinical problem-solving. (3) Skills proficiency ensuring healthcare workers master necessary skills to handle medical challenges presented in the simulation scenario. SimBegin mentors are trained to use Peyton’s method for design of effective skill training. (4) Simulation in teams, where healthcare workers can care for simulated patients in collaboration with others in a simulated relevant environment where they need to practise their knowledge and clinical skills. (5) Clinical care. Team simulations foster non-technical skills and are believed to be a driver for transformation of training learning outcomes into clinical care. By teaching SimBegin participants how to plan and run a pre-written scenario, do reflective debriefing, mentoring, and facilitator training SimBegin helps improve clinical performance, ensuring healthcare teams are better prepared to address medical challenges

The local facility champions facilitated continuous QI loops utilising simulation-based training following strategies illustrated in the Circle of Learning model (Fig. 2) [28]. The Circle of Learning strategies aim to “bridge cognitive and skill-based learning with real-life clinical experience” [23]. The model leans towards theories related to experiential learning, competency-based education, and training efficiency [29, 30]. Kolb is about experiential learning, and his circle is also appropriate to explain what we do in simulations (experience—reflection). Competency-based education is a movement away from exams (knowledge tests)—trainees are able to do things (master)—a combination of knowledge, skills, and attitude.

QI areas were identified through weekly review of the SBBC facilities’ own clinical data. A list of 52 quality indicators was provided every week and discussed in facility champion or national facilitator-led debriefing sessions using a deliberate practice approach. Deliberate practice aims to improve individual or team performance through intentional, goal-directed practice [31]. Through these discussions, clinical gaps were identified and categorised into sections: knowledge, individual skills, or team skills. Appropriate training interventions were planned, executed, and translated into clinical care.

### SBBC innovative tools

SBBC introduced several innovative tools designed to improve quality of care and to support the training interventions. The innovations were co-created by clinicians in Tanzania, researchers and engineers from Norway, and produced by Laerdal Global Health, Stavanger, Norway.

#### Clinical tools

Moyo is a foetal heart rate monitor designed for intermittent or continuous monitoring. Moyo aims to detect foetal distress, to support timely decision-making, and to reduce midwives’ workload [32–34]. NeoBeat is a newborn heart rate monitor designed to detect heart rate, guiding resuscitation attempts, and to support timely decision-making [35]. The Upright bag is a vertical bag-mask newborn ventilation device designed to improve mask seal and ventilation quality [36].

#### Training tools

The NeoNatalie Live simulator is a newborn resuscitation simulator that provides feedback on the quality of ventilation, airway management, and time to first ventilation. The simulator records and uploads all training activities to a database [18]. MamaNatalie is a wearable simulator designed to practise the third stage of labour and complications like retained placenta and PPH [21]. Both simulators were used for individual skill training and team simulations. No clinical tools were introduced for the maternal population, only the novel training component and the data-driven QI.

### Sustainability and scalability

A close collaboration with local, regional, and national health authorities was established before the start of the program. The SBBC tools were distributed to all included facilities, and all training content aligned with national obstetric and newborn care guidelines. Each SBBC facility established a dedicated “training corner” in the labour ward. A mentorship program, aiming to develop and support the national facilitators continuously, was established. Mentorship was led by international simulation experts from SAFER. The facility champions and the maternity ward staff at the facilities received supervision visits from the national facilitators every third month, where they received mentoring on clinical topics, clinical data, training, simulation, and the facilitator role.

### Data collection and management

This study utilises observational data from March 01, 2021, through December 31, 2023, from the 30 SBBC sites. The overall data collection and management is described in the SBBC study protocol and the primary paper [22, 23]. Every healthcare facility had two data collectors collecting routine provider registered clinical data on a daily basis by using a case report file installed on mobile phones or tablets. Pre-implementation data collection started March 1, 2021, at all sites. The start of SBBC implementation in the different regions is indicated in Table 1.

**Table 1:**
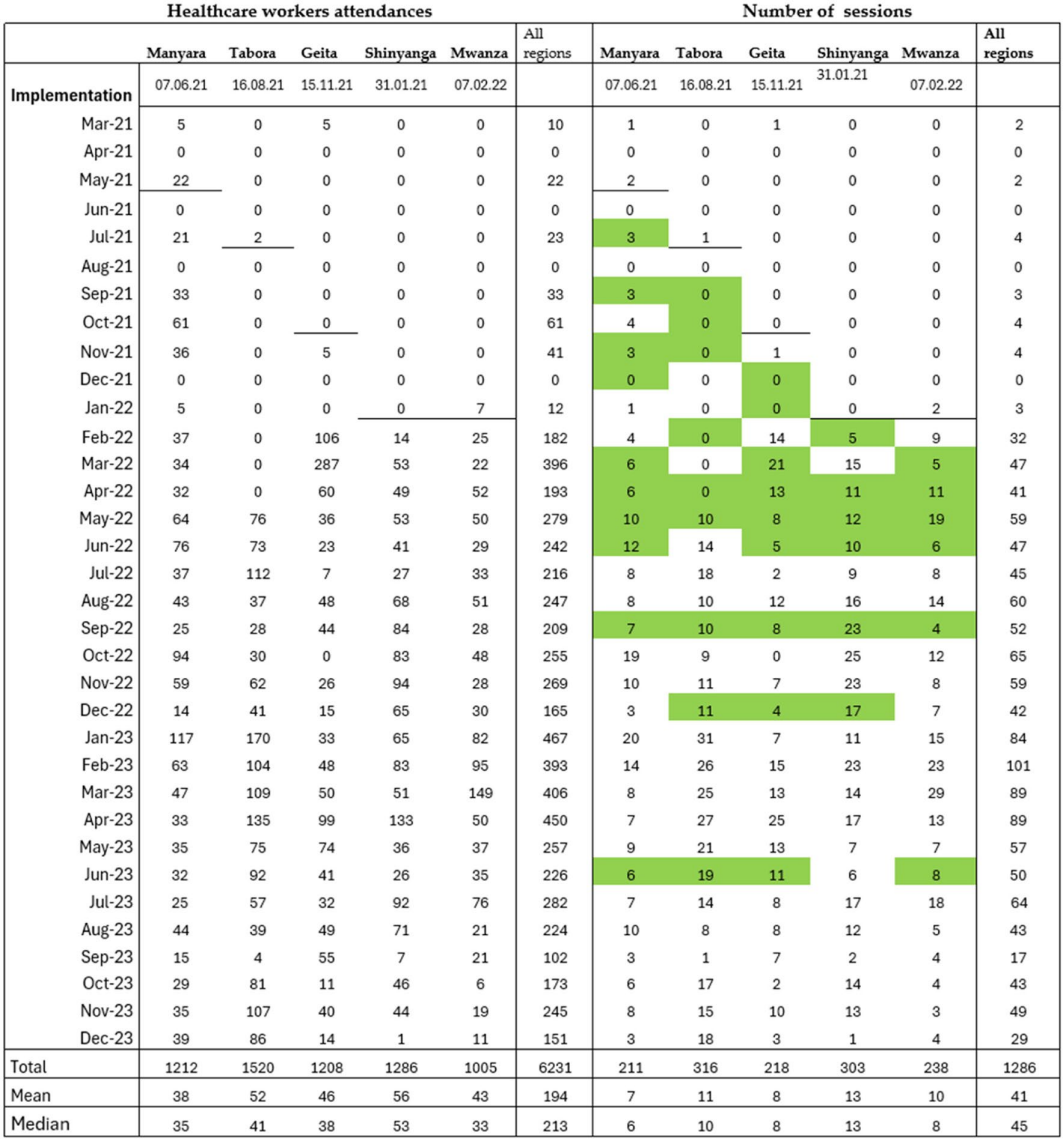
Number of healthcare workers trained and team simulation sessions per region

The baseline period started in March 2021 in all regions. The bold black lines indicate the start of SBBC implementation in the different regions following the step-wedged design. Manyara started implementation in June 2021. Uptake of facilitator-led team simulation sessions varied between regions, as indicated by the numbers. Months with supervision visits/mentoring are marked in green

The facility champions documented training activities in a facility-based training database on a regular basis. These data provided information about the simulation sessions. The database also documented how simulation training activities and continuous QI processes were implemented in the SBBC facilities.

### Statistical analysis

Numerical data were presented as numbers, means with standard deviations, and medians, and categorical data as numbers and proportions. The main analysis objective was to study the association between the number of trainings 1 month and maternal deaths the following month. A Poisson regression mixed model approach was used to analyse this relationship over time, taking into account dependency within regions and facilities. A logarithm transformation of the number of trainings was used for appropriate model fit. Furthermore, an ordinary Poisson regression model with period (1, 2, and 3) as a categorical predictor variable was used to model the change in total number of trainings in the three periods following the introduction of SimBegin® levels 1, 2, and 3, respectively. SPSS version 29.0.1.0 (171) and R version 4.3.3 were used for statistical analysis. A *P* value ≤ 0.05 was considered significant.

## Results

Following the initial SimBegin® training of the 15 national facilitators and facility champions, 464 HCWs participated in frequent and regular simulation sessions, referred to as recurrent participants. The simulation sessions described included all sessions regardless of medical topic. A total of 281,165 parturient women were enrolled in the study, 63,868 in baseline and 217,297 after start SBBC. The total number of maternal deaths was 291, i.e. 152 (240/100,000 births) in baseline and 139 (60/100,000 births) after start SBBC. Table 2 describes the incidence of maternal deaths at a regional level.

**Table 2:** Study periods, included women, and maternal deaths by region

**Region**		**Time period**		**Included women**	**Missing outcome data**	**Maternal deaths**	**Maternal survival**
**Manyara**	Baseline	01.03.21	06.06.21	3928	438 (11.2)	6 (0.15)	3484 (88.7)
	Implementation	07.06.21	31.12.23	36,449	114 (0.31)	12 (0.03)	36,323 (99.65)
**Tabora**	Baseline	01.03.21	15.08.21	6497	56 (0.86)	9 (0.14)	6432 (99)
	Implementation	16.08.21	31.12.23	42,190	265 (0.63)	54 (0.13)	41,871 (99.24)
**Geita**	Baseline	01.03.21	14.11.21	17,869	60 (0.34)	28 (0.56)	17,781 (99.51)
	Implementation	15.11.21	31.12.23	63,137	35 (0.06)	27 (0.04)	63,075 (99.90)
**Shinyanga**	Baseline	01.03.21	30.01.22	15,360	998 (6.5)	43 (0.28)	14,319 (93.22)
	Implementation	01.02.22	31.12.23	34,875	60 80.17)	22 (0.06)	34,793 (99.76)
**Mwanza**	Baseline	01.03.21	06.02.22	20,126	101 8 (0.50)	66 (0.33)	19,959 (99.17)
	Implementation	07.02.23	31.12.23	40,646	142 (0.35)	24 (0.06)	40,480 (99.59)
**All regions**	Baseline	01.03.21	06.02.22	63,868	1653 (2.6)	152 (0.24)	62,063 (97.17)
	Implementation	07.06.21	31.12.23	217,297	616 (0.28)	139 (0.06)	216,542 (99.65)
	Total	01.03.21	31.12.23	281,165	2269 (0.81)	291 (0.10)	278,605 (99.09)

Study periods for each region, number of included women in baseline and implementation, number of women with missing outcome information, and maternal deaths during baseline and after the start of implementation. Cases are shown as numbers (percentages)

### Training frequencies

After the start of SBBC implementation, 1220 simulation sessions featuring 6160 recurrent participants were documented (Table 1). A median of 186 (quartiles 102, 287) recurrent participants attended the simulation sessions per facility throughout the implementation period. The simulation sessions had an overall average attendance of 5 participants per session. A total of 591/1286 (46%) of all the simulation sessions had an HMS topic, focusing on management of PPH (*n* = 283), antepartum bleeding (*n* = 3), active management of the third stage of labour (*n* = 252), or eclampsia (*n* = 53). In total, 3293/6231 (53%) recurrent participants attended the HMS simulation sessions. This translates to an average attendance of 7 HMS-related sessions per healthcare worker during the implementation period.

**Fig. 3:**
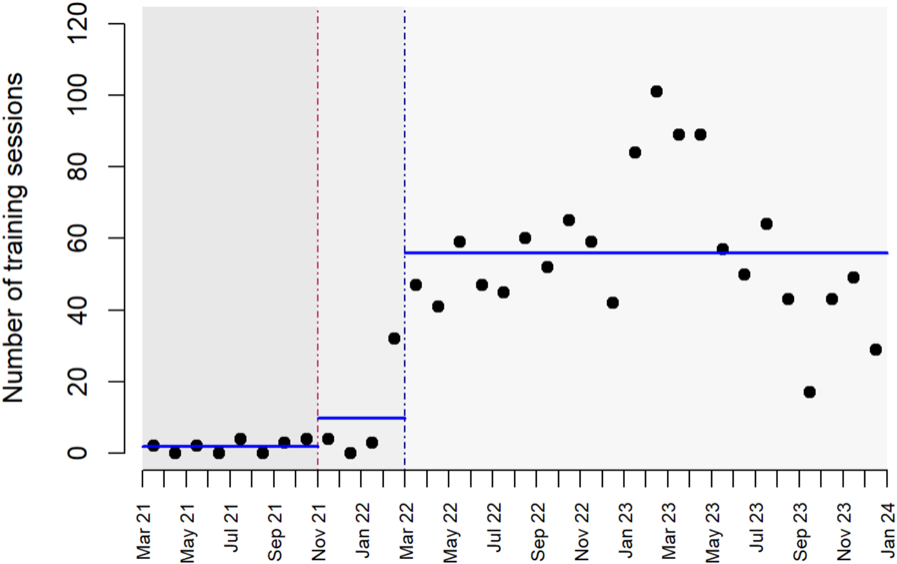
Introduction of the different SimBegin levels and number of team simulation sessions in all regions. At the start of SBBC implementation in March 2021, all the 15 selected facilitators were trained in SimBegin level 1 (green stippled line). SimBegin level 2 training was provided to all the 15 facilitators in November 2021 (red stippled line), and in March 2022, 8/15 facilitators were trained in level 3 (blue stippled line). Every dot represents the overall number of in situ team simulation training sessions for consecutive months

The overall frequency of simulation sessions increased significantly after the implementation of the different levels of SimBegin®, i.e. an average of 1.9, 9.8, and 56 trainings/month (*P* < 0.001) were documented after the introduction of levels 1, 2, and 3, respectively (Fig. 3). On average, 2 (SD 3) simulation sessions and 8 (SD 15) participants were documented per facility per month. However, there were large differences between the facilities, illustrated by a median of 0 (quartiles 0, 2) simulation sessions per month.

Time from the initial SBBC training (start of implementation) in the regions to uptake of regular training within the facilities varied. Manyara, Geita, Shinyanga, and Mwanza started simulation trainings immediately after SBBC implementation, while Tabora started simulation trainings in January 2022, 5 months post-implementation (Table 1). In Manyara, Geita, Shinyanga, and Mwanza, there seems to be an increase in training frequency following the mentoring visits. In Tabora, an increase can be seen after five mentoring visits. Shinyanga had the highest average of (*n* = 56) monthly recurrent participants and simulation sessions (*n* = 13) (Table 1).

### Association between training frequency and maternal deaths

**Fig. 4:**
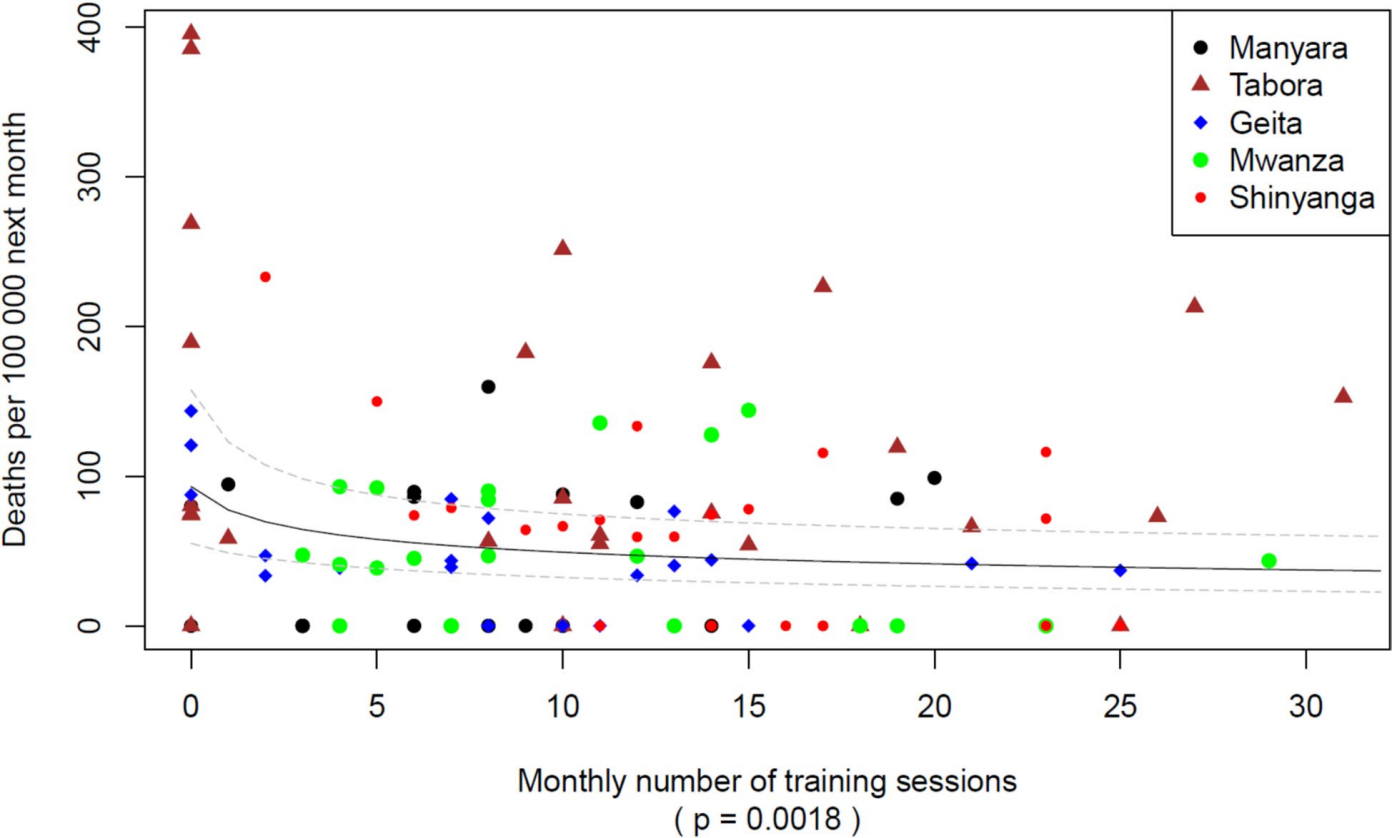
Association between maternal deaths and by region number of facilitator-led team simulations. The figure presents the number of all team simulation sessions (both newborn resuscitation and maternal emergencies) by region versus maternal deaths the following month. To compare training frequency in one period with outcomes in the following period, months provide an appropriate balance to do so. The lines in the figures represents the “expected number of deaths per 100,000” as a function of the number of trainings. The solid grey line illustrates the estimated logarithmic association between the number of training sessions per region per month and expected maternal deaths the following month, with pointwise 95% confidence intervals added as dashed lines. There are 6 facilities in each region, thus a value of 30 trainings on the x-axis translates to an average of 5 trainings per facility per month. A value of 6 on the x-axis translates to an average of 1 training per facility per month

**Fig. 5:**
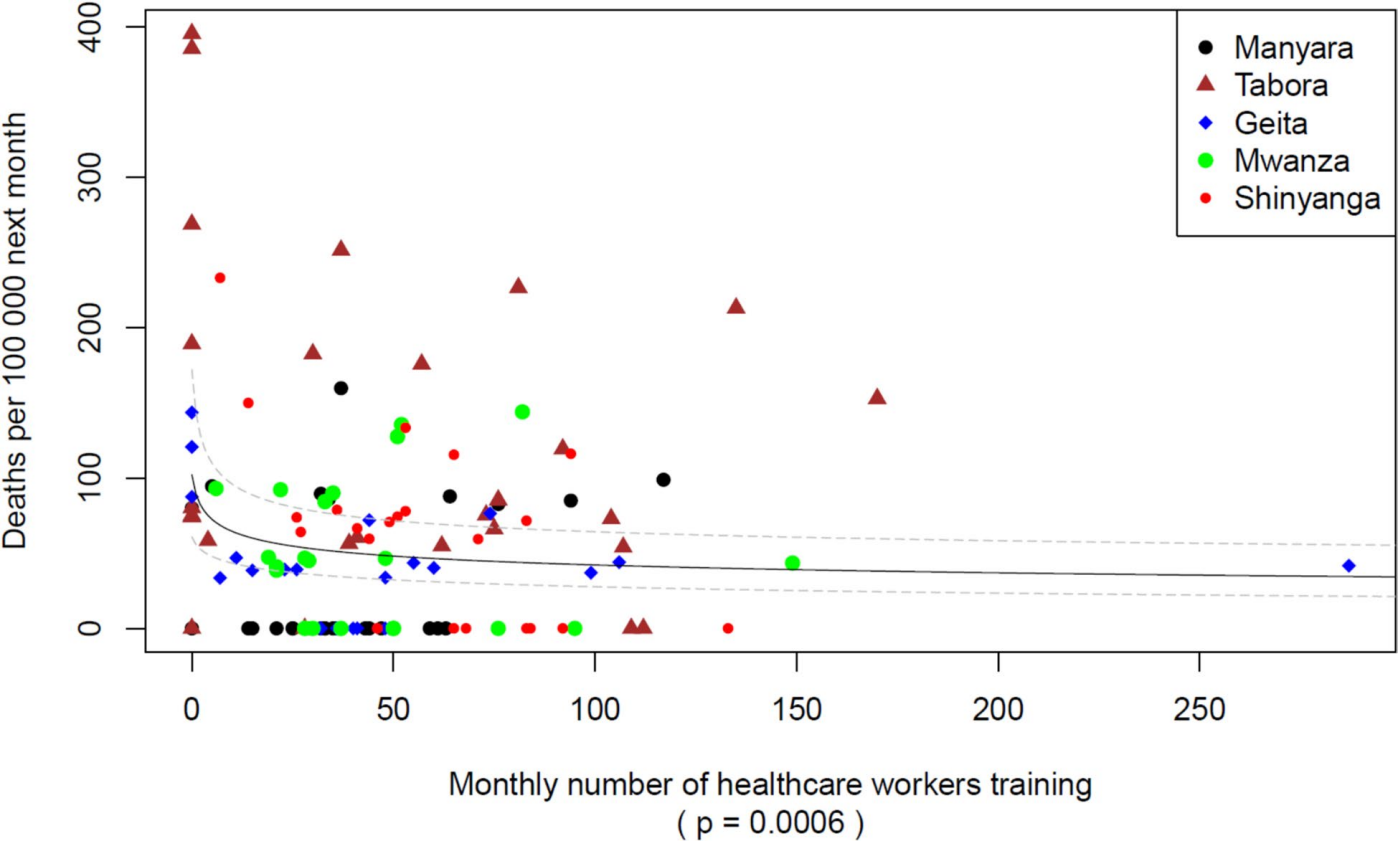
Association between maternal deaths and by region number of recurrent participants in these sessions. The figure presents the number of recurrent participants in the team simulation sessions (both newborn resuscitation and maternal emergencies) by region versus maternal deaths the following month. The solid grey line illustrates the estimated logarithmic association between the number of recurrent participants per region per month and expected maternal deaths the following month, with pointwise 95% confidence intervals added as dashed lines. There are 6 facilities in each region, thus a value of 30 healthcare workers on the x-axis translates to an average of 5 recurrent participants per facility per month

Figures 4 and 5 show the associations between the frequency of simulation sessions (Fig. 4) and the number of recurrent participants (Fig. 5) and maternal deaths the following month. For every one unit increase in the number of training sessions on a log scale, the risk of maternal death next month decreased by 23% (risk ratio exp(−0.257) = 0.77; *P* = 0.0018). For every one unit increase in the number of recurrent participants on a log scale, the risk of maternal death next month decreased by 16% (risk ratio exp(−0.16978) = 0.84; *P* = 0.0006).

## Discussion

In this study, we document significant associations between the frequency of simulation sessions, the number of recurring participants, and reduction in maternal deaths the following month. An overall reduction of 75% in maternal deaths from baseline to after the implementation of SBBC has been reported [23], and we report a large increase in the number of simulation sessions over the same period. The training activities following the introduction of this program were the only intervention targeting the maternal population, and we document a 23% and 16% reduced risk of maternal deaths the following month if the number of simulation sessions and recurrent participants, respectively, are increased by one unit on a logarithmic scale. An increase of one unit on the log scale would correspond to, for instance, increasing from 1 to 3 trainings or from 4 to 11 trainings or going from 10 to 27 participants or from 20 to 54 participants.

### Frequency of simulation sessions

This study documents a large number of simulation sessions following the introduction of the SimBegin® program. To the best of our knowledge, no other comparable program has previously reported—such numbers of simulation trainings over time in a low-resource context. Despite a high burden of work for each of the 464 healthcare workers working in the 30 SBBC facilities at any time, the simulation sessions involved 6231 recurring participants over the 24–32 months of SBBC implementation. This is particularly interesting, given the very low provider-patient ratio typical for these contexts. There are some variations in the uptake and implementation of simulation training across the regions and between the facilities, but a previous SBBC study did not document any association between a high burden of work and simulation training frequencies in the various SBBC sites [37].

We designed the SimBegin® facilitator training program based on existing evidence and previous practices. However, we did not have any experience in implementing simulation training in resource-challenged contexts where few healthcare workers had no prior exposure to simulation or reflection-based debriefings. Our previous experiences from facilitator courses and faculty development programs indicated that a common stand-alone train-the-facilitator training would not be sufficient to start simulation trainings. Therefore, an implementation strategy was designed for this purpose, and Fig. 3 demonstrates how the number of simulation sessions increased after conducting SimBegin® level 2 and level 3 trainings of selected facilitators. By this, we demonstrate the time, efforts, and follow-up required to establish a system supporting frequent simulation training. The 15 national facilitators had stakeholders’ support from the Tanzanian Ministry of Health, professional bodies, regional and local healthcare authorities as well as local institutional support. They received mentoring and more advanced training (including advanced debriefing techniques, human factors/cognitive skills and social skills, and scenario design) from simulation experts over time. As a result, their competency and confidence likely improved and benefitted the entire training cascade, leading to more frequent simulation training [23, 24]. We observed a decrease in the frequency of simulation sessions towards the end of the study period, and this is a concern that requires follow-up. The SBBC program is currently scaled up to 150 facilities, including a continuation of the initial 30 sites, allowing for further monitoring and evaluation over time.

If the goal is to use simulation methodology appropriately and to improve the quality of care, a current recommendation is to train those who are involved in facilitating simulation activities [17]. The 2023 Association for Simulated Practice in Healthcare (ASPIH) standard states that facilitators should receive facilitator training, learning how to establish psychological safety and conduct debriefing [38]. Due to limited resources, we were not able to formally train all the local facility champions as level 1 facilitators through the SimBegin® program. However, the national facilitators reported that the champions seemed to have learned by participating in simulation sessions conducted by the national facilitators during mentoring visits. This learning methodology is well known and described by Bandura [39]. We believe that the limited number of pre-written scenarios available in the facilities (only four in the initial phase) in combination with the easy-to-follow SimBegin® debriefing framework (named CORE) and printed cue cards used as cognitive aids made it easier for the champions to learn how to run simulation sessions and structured reflection-driven debriefings. Since this learning process did not follow a specific structure or schedule, it might have contributed to what seems to be a slow uptake following the introduction of different levels of SimBegin®, as indicated in Fig. 3 and Table 1.

Despite the high number of simulation sessions reported in this study, we still think that there is unreleased potential and that the number of sessions could increase. Previous research from high-resource contexts have found weekly simulation training to be feasible and to improve patient outcomes [40, 41]. We believe that this frequency may be possible also in a low-resource context. As indicated in Table 1, we were able to reach an average of 2 (SD 3) team simulations and 8 (SD 15) participants per month across the 30 SBBC sites. We believe that formal facilitator training will improve the competence and confidence of the facilitators and that again will foster motivation to conduct more simulation sessions. Future studies are needed to investigate these associations.

#### The association between frequency of simulation sessions and reduced maternal mortality

This is the first study to our knowledge demonstrating an association between the frequency of simulation sessions, the number of recurring participants, and the reduction in maternal mortality rates. The more simulation sessions conducted at the SBBC sites, the fewer mothers died the following month. The steep initial reduction in maternal deaths shows that the impact per added simulation session or participant is highest when the numbers of sessions or participants are low. Based on a visual inspection of the fitted lines, there seems to be little additional to gain beyond approximately 15 trainings and 100 participants, respectively.

We believe several factors enforced the impact of the simulation sessions. The systematic weekly review of our data with the healthcare workers might have contributed to ownership of performance gaps revealed during these discussions, thus motivating training aiming to close these gaps.

In addition to medical topics, the simulation sessions focused on team skills. All pre-written scenarios provided in the SBBC project had one team skill focused learning objective in addition to medical objectives. A series of studies from Brogaard and colleagues on obstetric emergencies found a significant association between clinical teams’ team (non-technical) skills and team performance regardless of the type of incident [42–45]. By strengthening the team (non-technical) skills of the clinical team, the clinical performance is also improved regardless of the nature of the medical emergency.

### Systematic QI efforts and the utilisation of the Circle of Learning as a QI tool

The SBBC training components and the systematic QI efforts were the only interventions in the SBBC bundle addressing maternal emergencies. The Circle of Learning was introduced as a translational tool, used to identify and categorise clinical gaps through a deliberate practice approach, and to use suitable training methods when designing training to close the gaps [46]. We investigated the association between the fourth (simulation in teams) and fifth component (clinical practice) in the CoL (Fig. 2) and the results indicate that the systematic efforts led to increased quality of care and improved patient outcomes [23]. Another SBBC study reported that frequent simulation trainings empowered midwives [47]. Through increased knowledge, skills, and confidence, the midwives were enabled to treat life-threatening conditions like PPH without having to wait for a physician to do the life-saving interventions like removal of a retained placenta. The training also triggered a cultural shift from a “blame and shame” into a more learning culture. The healthcare workers reported increased psychological safety [47]. A significant improvement in documentation of various patient indicators and outcomes was also found post SBBC implementation (Table 2) [48].

A newly published Cochrane review on implementation models to prevent, detect, and treat PPH could not document any strategies leading to improved maternal outcomes [49]. We believe the SBBC implementation model differs from the included studies in several ways, i.e. (1) proper training of facilitators through the SimBegin program, (2) leading to frequent simulation trainings followed by reflective debriefs, (3) with systematic data-driven QI efforts using the Circle of Learning approach led by the local staff, (4) with regular support and mentorship visits, and finally (5) close collaboration with national health authorities.

### Strengths and limitations

The large patient population, inclusion of different levels of health facilities (health centres, district and regional hospitals), a study period of 2–2.5 years, and a high number of team simulation sessions strengthen the findings in this study. The project had decision-makers’ endorsement from the Ministry of Health, local project lead, local follow-up, and mentoring. This facilitated a good overview and control of potential confounding non-SBBC interventions or trainings in the healthcare facilities during the data collection period. No such confounders were identified or reported.

Limitations of this study: The manual documentation in logbooks added an extra burden of work to the facility champions after conducting the simulation sessions, and we believe this might have led to missing documentation of trainings. We did not have a system to identify the individual simulation attendants, and we could not identify how many trainings each of the 464 healthcare workers attended as recurrent participants. Such information could have enabled further analyses of the association between individual exposure to team simulations and clinical impact.

## Conclusion

This study indicates that the introduction of the SimBegin® program and the implementation strategy led to frequent and regular simulation sessions in the 30 SBBC facilities. A higher frequency of simulation trainings and a number of recurrent participants were associated with reduced maternal deaths the following month. Further research is needed to investigate how the potential training capacity can be utilised more efficiently and how documentation of the training activities might improve.

## Supplementary Information

Supplementary Material 1.

Supplementary Material 2.

Supplementary Material 3.

Supplementary Material 4.

## Data Availability

The data that support the findings of this study are available from Haydom Lutheran Hospital, Research Department but restriction apply to the availability of these data, which were used under license for the current study, and so are not publicly available. Data are however available from the authors upon reasonable request and with permission of Haydom Lutheran Hospital, Research Department.

## Abbreviations

SBBC: Safer Births Bundle of Care
CQI: Continuous quality improvement
WHO: World Health Organization
QI: Quality improvement
PPH: Postpartum haemorrhage
TAMA: Tanzanian Midwifery Association (nevnt en gang, må vi ha med forkortelse?)
PAT: Pediatric Associations of Tanzania (nevnt en gang, må vi ha med forkortelse?)
AGOTA: Association of Obstetricians and Gynaecology (nevnt en gang, må vi ha med forkortelse?)
HBB: Helping Babies Breathe
HMS BAB: Helping Mothers Survive Bleeding after Birth
ASPIH: The Association for Simulated Practice in Healthcare
