# Supplementary material for: Frequency of team simulation and reduction in maternal deaths following Safer Births Bundle of Care implementation—a prospective observational study
Source: Adv Simul (Lond). 2025 Nov 14;10:56. doi: 10.1186/s41077-025-00387-7 (PMC12619334; doi:10.1186/s41077-025-00387-7)
Supplement: Supplementary file 1 — Supplementary Material 1. [file 41077_2025_387_MOESM1_ESM.pdf]

Scenario template

**Category:**	Newborn Health
**Theme:**	Resuscitation
**Learning objectives:**	1. Preparation of environment, equipment, personnel and mother for birth<br>2. Provide immediate newborn care at birth and identification of need and provision of appropriate intervention<br>3. Closed loop communication between the team members when sharing critical information<br>4. Respectful and supportive communication with the mother
**Events:**	Pregnant mother arrives at health facility for delivery in fully dilated condition. The participants are to identify that the birth is imminent, prepare the labour room, get the equipment readied and check the functionality. The baby is born and not breathing at birth. Participants are to provide immediate care at birth for the newborn (drying the baby) and identify the meconium blocking airway of baby and suction the airway, stimulating the baby by rubbing the back to help the baby breathe. Once the baby breathes, participants are to perform cord clamping and cutting, skin to skin care and communicate with the mother. Closed loop communication among team members is expected to be done throughout the scenario for critical information sharing.
**Action points:** (critical events in scenario)	• Preparation of environment, equipment (including checking for the functionality), personnel and mother for birth<br>• Care at birth for newborn<br>• Identification that the baby is not breathing<br>• Identification that the airway is filled with meconium and provide suctioning to help the baby breathe<br>• Closed loop communication among team members<br>• After the baby breathes, clamp and cut the cord, skin to skin care<br>• Communication with the mother in a respectful, empathetic and supportive manner.
**Patient behavior:** (for simulated patients or operators)	Anxious mother- worries about what happened to the baby.
**Patient description:** (background information for facilitator)	
**Information to participants:**	Maya Angelou, G3 P2 L2 (Gravida 3, para 2, living children 2), who has attended 5 ante-natal visits at your facility, has now arrived at your facility with full dilatation and almost ready to deliver. Her pregnancy was uneventful, and her previous deliveries were normal vaginal deliveries. You have the required logistics to conduct a normal delivery and immediate newborn care.<br><br>Participants:<br>1. Senior midwife<br>2. Junior midwife<br>3. Neonatal nurse

Scenario template

**SIM info:**	Type	Manikin (able to breathe, cry and has a heart rate- preferably Neonatalie Complete) with an operator in a labour room setting. The operator could be one of the faculty.
	Dressing	
	Medical equipment	• Delivery tray (2 artery forceps, umbilical cord cutting scissors, umbilical cord clamp, pads)<br>• a pair of warm towels to receive and dry the baby<br>• newborn suction bulb<br>• Stethoscope<br>• NeoBeat<br>• newborn bag and mask
	Medicine	
**Vital signs:**	Airway	Initially blocked with meconium
	Breathing	Not breathing
	Circulation	Heart rate 80 beats per minute
	Disability	
	Øvrige verdier	Baby is pink in colour and warm to touch

Changes in progress:

Vital signs	Expected actions from participants
Beginning of scenario<br>No breathing<br>Heart rate 80/min<br>Baby is pink in colour and warm to touch	A. Essential care for baby at birth<br>B. Recognizes that baby is not crying<br>C. Dries the baby<br>D. Positions baby's head<br>E. Identifies meconium blocking the airway and suctions<br>F. Stimulates the baby by rubbing the back of the baby<br>G. Maintain closed loop communication throughout the scenario
After the participants have done the expected actions A,B,C,D,E,F:<br><br>Heart rate improves to 100 beats/min baby starts breathing- 10 breaths per minute and increases to 40 breaths per minutes quickly	H. Identifies that baby is breathing spontaneously<br>I. Places the baby in skin to skin contact with the mother<br>J. Checks on the baby regularly<br>K. Communicates with mother in respectful and supportive manner

Keywords for debriefing:

Focus on learning objectives

What was successful, and what made it become a success?

What aspects would need to be improved and how?

References:

https://www.healthynewbornnetwork.org/hnn-content/uploads/HBB_Action-Plan_2016.pdf
http://gynecology.sbmu.ac.ir/uploads/4_5922741106407112942.pdf

©SAFER2018
