# Supplementary material for: Frequency of team simulation and reduction in maternal deaths following Safer Births Bundle of Care implementation—a prospective observational study
Source: Adv Simul (Lond). 2025 Nov 14;10:56. doi: 10.1186/s41077-025-00387-7 (PMC12619334; doi:10.1186/s41077-025-00387-7)
Supplement: Supplementary file 2 — Supplementary Material 2. [file 41077_2025_387_MOESM2_ESM.pdf]

Scenario template

**Category:**	Maternal Health
**Theme:**	Bleeding after Birth
**Learning objectives:**	1. Identifying and providing effective care for managing fully retained placenta<br>2. Closed-loop communication among team members to ensure shared situational awareness<br>3. Respectful and supportive communication with the mother and the family members
**Events:**	A mother (gravida 3 para 2 living babies 2, abortions 0 and 40 weeks gestation) gives birth, and the amniotic fluid is clear. The participants are expected to provide active management of third stage of labour, comforting the mother, participants are expected to identify the retained placenta and provide management for retained placenta while also comforting the mother.
**Action points:** (critical events in scenario)	• Identification of retained placenta<br>• Appropriate management of retained placenta- repeating 10IU Inj Oxytocin IM, emptying the bladder, controlled cord traction, Inj diazepam 10 mg IM, starting IV fluids, giving antibiotic- ampicillin or cefazolin, procedure of manual removal of placenta, when the placenta is removed- checking the completeness of placenta and ensuring there is a good uterine tone and that bleeding has stopped<br>• Closed loop communication among team members<br>• Respectful and supportive communication with the mother and family.
**Patient behavior: (**for simulated patients or operators)	Mother wearing the manikin at her waist: hold onto the placenta and don't release it after the AMTSL is performed. Wait for my cue before you release the placenta (I will raise my hand when I would want you to release the placenta). When there is much activity happening around you for removal of placenta- you become anxious and start asking questions frequently.<br><br>Anxious mother-in-law - worries about what happened to the daughter-in-law.
**Patient description:** (background information for facilitator)	Maya Angelou, G3 P2 L2 (Gravida 3, para 2, living children 2), who has attended 3 ante-natal visits at your facility, arrived at your facility with 40 weeks pregnancy (full term) full dilatation. She is delivering now. Her previous deliveries were normal vaginal deliveries.<br><br>Mother is wearing a traditional dress.
**Information to participants:**	Maya Angelou, G3 P2 L2 (Gravida 3, para 2, living children 2), who has attended 3 ante-natal visits at your facility, arrived at your facility with 40 weeks pregnancy (full term) full dilatation and is delivering now.<br><br>You have the required logistics to conduct a normal delivery and manage any common complications for the mother and baby post-partum. You have already identified a helper, prepared the labour room, washed your hands, and checked your equipment. The baby is born, and the amniotic fluid is clear. You have to manage the mother hereon.<br><br>Participants:<br>1. Senior midwife<br>2. Junior midwife<br>3. Junior doctor / junior nurse (according to availability)

©SAFER2018

Scenario template

**SIM info:**	Type	Manikin tied around the waist of the operator (the mother)- which can show uterine bleed, uterine tone and has a placenta (preferably MamaNatalie) in a labour room setting.
	Dressing	Mother with the manikin tied to her abdomen (preferably the mother is one of the faculty)
	Medical equipment	• Delivery tray (2 artery forceps, umbilical cord cutting scissors, umbilical cord clamp, pads)<br>• a pair of warm towels to receive and dry the baby<br>• long gloves<br>• IV cannula (18 guage) and IV set<br>• IV fluid (normal saline)<br>• Gauze pads<br>• newborn suction bulb<br>• stethoscope/ NeoBeat<br>• newborn bag and mask<br>• radiant warmer/ adequately established ventilation area
	Medicine	• Inj Oxytocin with syringe<br>• Inj Diazepam<br>• Inj Ampicillin/ Cefazolin<br>• Inj. Adrenalin
**Vital signs:**	Airway	Open
	Breathing	18 per minute
	Circulation	Heart rate 100 per minute
	Disability	none
	Øvrige verdier	

Changes in progress:

Vital signs	Expected participant actions
At beginning of scenario:<br>Heart rate 100/min<br>Breathing: 18/ minute<br>Mother is conscious and able to talk and respond well	A. Provides active management of third stage of labour<br>B. Communicates with the mother
At 30 seconds into scenario: announce that it is 1 hour after birth<br>Heart rate 110/min<br>Breathing: 20/min<br>Mother is conscious but anxious	A. Identifies that this is a case of retained placenta<br>B. Communicates with the mother<br>C. Administers 10 IU Oxytocin IM<br>D. Controlled cord traction<br>E. Administers 10 mg Diazepam IM<br>F. Inserts IV cannula and starts IV fluid/ NS<br>G. Dons long gloves and performs the procedure for manual removal of placenta<br>H. After the placenta is removed, checks the completeness of the placenta<br>I. Checks the uterine tone and bleeding<br>J. Comforts the mother<br>K. Closed loop communication among the team members is taking place through-out the scenario

©SAFER2018

**Keywords for debriefing:**	Focus on learning objectives – especially clear and confirming communication<br><br>1. How did you identify that it was a case of retained placenta? (decision making)<br>2. Were you able to follow the Action Plan? (knowledge acquisition and skills practice)<br>3. Have you encountered similar cases in your practice? (application)<br>3. What went well and what could have gone better?<br>4. What did you learn?<br>5. What will you do differently next time?
**References:**	https://reprolineplus.org/resources/HMS-English<br><br>https://apps.who.int/iris/bitstream/handle/10665/75411/9789241548502_eng.pdf;jsessionid=5A03B41C25DE880C71BC61F4E4C12420?sequence=1

©SAFER2018
