# Supplementary material for: Frequency of team simulation and reduction in maternal deaths following Safer Births Bundle of Care implementation—a prospective observational study
Source: Adv Simul (Lond). 2025 Nov 14;10:56. doi: 10.1186/s41077-025-00387-7 (PMC12619334; doi:10.1186/s41077-025-00387-7)
Supplement: Supplementary file 3 — Supplementary Material 3. [file 41077_2025_387_MOESM3_ESM.pdf]

Scenario template

**Category:**	Newborn Health
**Theme:**	Resuscitation
**Learning objectives:**	1. Providing effective newborn resuscitation (including bag mask ventilation)<br>2. Closed loop communication among team members<br>3. Provides clinical leadership by giving clear instructions, being audible and visible
**Events:**	A baby (34 weeks gestation) is born, and the amniotic fluid is clear. The participants are expected to provide immediate newborn care, identify that the baby is not crying, stimulate the baby by rubbing the back of the baby, cuts the cord and move the baby to the ventilation area and start effective bag mask ventilation. The chest rise is seen after few ventilations and the heart rate picks up after a minute of ventilation. The baby starts breathing after 3 minutes of effective bag mask ventilation- participants are expected to identify the same and stop ventilating and observe the baby. Closed loop communication among team members and clinical leadership by one of the team members.
**Action points:** (critical events in scenario)	• Identification that the baby is not breathing and resuscitate the baby<br>• Identify spontaneous breathing of the baby to stop ventilation and observation of the baby<br>• Appropriate communication with the mother.
**Patient behavior:** (for simulated patients or operators)	Anxious mother- worries about what happened to the baby.
**Patient description:** (background information for facilitator)	
**Information to participants:**	Maya Angelou, G3 P2 L2 (Gravida 3, para 2, living children 2), who has attended 3 ante-natal visits at your facility, arrived at your facility with 34 weeks pregnancy (7.5 months) full dilatation and almost ready to deliver. Her previous deliveries were normal vaginal deliveries. You have the required logistics to conduct a normal delivery and immediate newborn care. You have already identified a helper, prepared an area for ventilation, washed your hands, and checked your equipment. The baby is born, and the amniotic fluid is clear.

©SAFER2018

Scenario template

**SIM info:**	Type	Manikin (able to breathe, cry and has a heart rate- possibly NeoNatalie complete) with an operator (preferably the mother as an actor) in a labour room setting
	Dressing	
	Medical equipment	• Delivery tray (2 artery forceps, umbilical cord cutting scissors, umbilical cord clamp, pads)<br>• a pair of warm towels to receive and dry the baby<br>• newborn suction bulb<br>• stethoscope<br>• NeoBeat<br>• newborn bag and mask<br>• radiant warmer/ adequately established ventilation area
	Medicine	Adrenalin
**Vital signs:**	Airway	Secretions in the mouth
	Breathing	Not breathing
	Circulation	Heart rate 60 beats per minute
	Disability	
	Øvrige verdier	Baby is pale in colour and slightly warm to touch

**Changes in progress:**	Vital signs	Expected participant actions
	At beginning of scenario:<br>Heart rate 60/min<br>No breathing<br>Baby pale in colour and warm to touch	A. Recognizes that baby is not crying and calls for help<br>B. Dries the baby<br>C. Positions baby's head<br>D. Stimulates the baby by rubbing the back of the baby, suctioning to remove secretions in the mouth<br>E. Identifies that the chest rise is not there- clamps and cuts the umbilical cord and moves the baby to ventilation area
	At 30 seconds into scenario:<br>Heart rate 60/min<br>No breathing<br>Baby pale in colour and warm to touch	Starts bag mask ventilation- identifies that there is no chest rise even after few ventilations- re-positions the baby and re-applies the mask to ensure better seal- gets a chest rise with ventilation
	After 1 minute of effective ventilation, heart rate becomes 80 /min	Ventilation to continue
	After 2.5 minutes of effective ventilation, heart rate improves to 100 beats/min<br>Babies spontaneous respiration starts at 10 breaths per minute	Ventilation to continue. Identify spontaneous respiration has started and assesses respiration
	After 3 minutes, heart rate is 120/min and spontaneous respiration is 40 breaths per minute	Identifies spontaneous respiration and stops ventilation
		Observes baby's heart rate, respiration and colour.<br>Closed loop communication among team members and clinical leadership seen throughout the scenario

©SAFER2018

Scenario template

**Keywords for debriefing:**	Focus on learning objectives – especially clear and confirming communication<br><br>1. What happened when the baby was born?<br>2. Did you follow the Action Plan?<br>3. What went well and what could have gone better?<br>4. What did you learn and what would you want to change if you were to repeat the same scenario over again?<br>5. What will you do differently next time?
**References:**	https://www.healthynewbornnetwork.org/hnn-content/uploads/HBB_Action-Plan_2016.pdf<br>http://gynecology.sbmu.ac.ir/uploads/4_5922741106407112942.pdf

©SAFER2018
