# Supplementary material for: Frequency of team simulation and reduction in maternal deaths following Safer Births Bundle of Care implementation—a prospective observational study
Source: Adv Simul (Lond). 2025 Nov 14;10:56. doi: 10.1186/s41077-025-00387-7 (PMC12619334; doi:10.1186/s41077-025-00387-7)
Supplement: Supplementary file 4 — Supplementary Material 4. [file 41077_2025_387_MOESM4_ESM.pdf]

# Scenario template

| | |
|---|---|
| **Category:** | Maternal Health |
| **Theme:** | Bleeding after Birth |
| **Learning objectives:** | 1. Providing effective care for managing bleeding after birth<br>2. Closed-loop communication among team members to ensure shared situational awareness<br>3. Respectful and supportive communication with the mother and the family members |
| | |
| **Events:** | A mother (gravida 4 para 3 living babies 3, abortions 0 and 40 weeks gestation) gives birth, and the amniotic fluid is clear. The participants are expected to identify atonic uterus, bleeding after birth, provide uterine massage, medication for controlling the uterine bleeding and bimanual compression while communicating with the mother and the family. |
| | |
| **Action points:**<br>(critical events in scenario) | • Identification of atonic uterus and bleeding after birth<br>• Appropriate management of bleeding after birth-<br>  o Calling for help<br>  o Massaging the uterus<br>  o Administering 10 IU Inj oxytocin IM at 60 drops per minute or 800 mcg misoprostol orally or sublingually<br>  o Regular checking of uterine tone and bleeding<br>  o Checking and catheterizing bladder<br>  o Inspects the placenta to ensure completeness<br>  o Starting IV infusion with 20IU Inj oxytocin in 1 litre Ringer Lactate/ other IV fluid<br>  o Collects blood for Hb, bedside clotting tests and cross matching if blood can be made available<br>  o Administer 1gm IV Tranexemic Acid diluted in 10 ml distilled water and given over 10 minutes<br>  o Provides bi-manual compression<br>  o Provides respectful maternity care<br>• Appropriate communication with the mother. |
| | |
| **Patient behavior:**<br>(for simulated patients or operators) | Mother wearing the manikin at her waist: Keep the uterine tone atonic and start bleeding at the beginning of the scenario and don’t stop until you get a cue to stop the bleed. When there is much activity happening around- you become anxious and ask questions and complain of dizziness.<br><br>Anxious husband - worries about what happened to wife. |
| | |
| **Patient description:**<br>(background information for facilitator) | Maya Angelou, G3 P2 L2 (Gravida 4, para 3, living children 3), who has attended 3 ante-natal visits at the facility, arrived at the facility with 40 weeks pregnancy (full term) full dilatation and delivered normally. Her previous deliveries were normal vaginal deliveries too. AMTSL (active management of third stage of labour) has been performed already. |
| | |
| **Information to participants:** | Maya Angelou, G3 P2 L2 (Gravida 4, para 3, living children 3), who has attended 3 ante-natal visits at your facility, arrived at your facility with 40 weeks pregnancy (full term) full dilatation and you have helped her deliver normally and performed AMTSL.<br><br>You have the required logistics to manage any common complications for the mother and baby post-partum. You have already identified a helper, prepared the labour room, washed your hands, and checked your equipment.<br><br>Participants:<br>1. Senior midwife<br>2. Junior midwife<br>3. Junior doctor<br>4. Junior nurse from emergency room |

©SAFER2018

# Scenario template

SAFER

| | | |
|---|---|---|
| **SIM info:** | Type | Manikin tied around the waist of the operator (the mother)- which can show uterine bleed, uterine tone and has a placenta (preferably MamaNatalie) in a labour room setting |
| | Dressing | Mother with the manikin tied to her abdomen |
| | Medical equipment | • Delivery tray (2 artery forceps, umbilical cord cutting scissors, umbilical cord clamp, pads) a pair of warm towels to receive and dry the baby, newborn suction bulb<br>• long gloves<br>• IV cannula (18 guage) and IV set<br>• IV fluid (normal saline/ ringers lactate)<br>• Gauze pads<br>• stethoscope/ NeoBeat<br>• newborn bag and mask<br>• radiant warmer/ adequately established ventilation area |
| | Medicine | • Inj Oxytocin with syringe<br>• Inj Diazepam<br>• Inj Ampicillin/ Cefazolin<br>• Inj. Adrenalin<br>• Tab misoprostol |
| | | |
| **Vital signs:** | Airway | Open |
| | Breathing | 18 per minute |
| | Circulation | Heart rate 100 per minute |
| | Disability | none |
| | Øvrige verdier | |
| | | |

| | | |
|---|---|---|
| **Changes in progress:** | Vital signs | Expected participant actions |
| | At beginning of scenario:<br>Heart rate 100/min<br>BP 120/80 mmHg<br>Breathing: 18/ minute<br>Heavy uterine bleeding<br>Mother is conscious and able to talk and respond well | A. Identifies this is a case of atonic uterus leading to bleeding after birth<br>B. Calls for help<br>C. Starts uterine massage<br>D. Checks bleeding and uterine tone regularly<br>E. Measures vitals<br>F. Communicates with the mother |
| | At 1 minute into scenario:<br>Heart rate 110/min<br>BP 110/70 mmHg<br>Breathing: 20/min<br>Heavy uterine bleeding<br>Mother is conscious, starts sweating, anxious | A. Administers 10IU Inj Oxytocin IM<br>B. Catheterizes bladder<br>C. Checks placenta for any missing pieces<br>D. Inserts IV cannula<br>E. Starts IV infusion of NS/ ringerslactate with 20 IU Inj Oxytocin at 60 drops per minute<br>F. Checks bleeding and uterine tone regularly<br>G. Measures vitals<br>H. Communicates with the mother<br>I. Collects blood for HB, Bedside clotting tests and grouping and cross matching in case blood is available |
| | At 3 minutes into scenario<br>Heart rate 130/min<br>BP 100/60 mmHg | A. Administers Inj Tranaxemic Acid 1 gm IV in 10 ml distilled water over 10 minutes |

©SAFER2018

| | | |
|---|---|---|
| | Breathing 22/min<br>Heavy uterine bleeding<br>Cold and clammy skin, sweating and mother complains of dizziness<br><br>After bimanual uterine compression is done, uterine bleeding reduces and finally stops | B. Continue IV infusion of IV fluid with oxytocin at 60 drops per minute<br>C. Checks bleeding and uterine tone regularly<br>D. Measures vitals<br>E. Communicates with the mother<br>F. Washes hands, puts on long gloves and performs bimanual uterine compression |
| | | |
| **Keywords for debriefing:** | Focus on learning objectives – especially clear and confirming communication<br><br>1. How did you identify that this was a case of post-partum haemorrhage due to atonic uterus? (decision making)<br>2. Were you able to follow the Action Plan? (knowledge and skills)<br>3. What went well and what could have been done better?<br>4. What did you learn?<br>5. What will you do differently next time? | |
| | | |
| **References:** | https://reprolineplus.org/resources/HMS-English<br><br>https://apps.who.int/iris/bitstream/handle/10665/75411/9789241548502_eng.pdf;jsessionid=5A03B41C25DE880C71BC61F4E4C12420?sequence=1 | |

©SAFER2018
